# Mandatory minimum sentencing policies and cocaine use in the U.S., 1985–2013

**DOI:** 10.1186/s12914-018-0182-2

**Published:** 2018-11-29

**Authors:** Lauryn Saxe Walker, Briana Mezuk

**Affiliations:** 1 Department of Health Behavior and Policy, Virginia Commonwealth University School of Medicine, 830 E. Main St, Richmond, VA USA; 2 Department of Epidemiology, University of Michigan School of Public Health, 1415 Washington Heights, Ann Arbor, MI USA; 3 Institute for Social Research, University of Michigan, 426 Thompson St, Ann Arbor, MI USA; 4 Division of Epidemiology, Department of Family Medicine and Population Health, Virginia Commonwealth University School of Medicine, 830 E. Main St, Richmond, VA USA

**Keywords:** Substance abuse, Substance use disorder, Mandatory minimum sentencing, Cocaine use, Crack cocaine, Anti-drug abuse act, Fair sentencing act, Freebase cocaine, Cocaine base

## Abstract

**Background:**

As of May 2017, the United States federal government renewed its prioritization for the enforcement of mandatory minimum sentences for illicit drug offenses. While the effect of such policies on racial disparities in incarceration is well-documented, less is known about the extent to which these laws are associated with decreased drug use. This study aims to identify changes in cocaine use associated with mandatory minimum sentencing policies by examining differential sentences for powder and crack cocaine set by the Anti-Drug Abuse Act (ADAA) (100:1) and the Fair Sentencing Act (FSA), which reduced the disparate sentencing to 18:1.

**Methods:**

Using data from National Survey on Drug Use and Health, we examined past-year cocaine use before and after implementation of the ADAA (1985–1990, *N* = 21,296) and FSA (2009–2013, *N* = 130,574). We used weighted logistic regressions and Z-tests across models to identify differential change in use between crack and powder cocaine. Prescription drug misuse, or use outside prescribed indication or dose, was modeled as a negative control to identify underlying drug trends not related to sentencing policies.

**Results:**

Despite harsher ADAA penalties for crack compared to powder cocaine, there was no decrease in crack use following implementation of sentencing policies (odds ratio (OR): 0.72, *p* = 0.13), although both powder cocaine use and misuse of prescription drugs (the negative control) decreased (OR: 0.59, *p* < 0.01; OR: 0.42, *p* < 0.01 respectively). Furthermore, there was no change in crack use following the FSA, but powder cocaine use decreased, despite no changes to powder cocaine sentences (OR: 0.81, *p* = 0.02), suggesting that drug use is driven by factors not associated with sentencing policy.

**Conclusions:**

Despite harsher penalties for crack versus powder cocaine, crack use declined less than powder cocaine and even less than drugs not included in sentencing policies. These findings suggest that mandatory minimum sentencing may not be an effective method of deterring cocaine use.

## Background

In May 2017, US Attorney General Jeffrey Sessions sent a memorandum directing federal prosecutors that they “must disclose to the sentencing court all facts that impact the sentencing guidelines or mandatory minimum sentences [1].” With this direction, a renewed emphasis was placed on mandatory sentences, reversing a 2013 policy that permitted prosecutors to withhold information to avoid triggering mandatory sentencing guidelines [1]. Mandatory minimum sentences have been used for centuries to target crimes seen as especially serious or disruptive [2]. However, the efficacy of these laws in reducing targeted behaviors remains unclear [2–4]. The handful of rigorous evaluations conducted to date have focused on firearms, and have demonstrated little effect of mandatory minimum sentences on gun crimes [3, 5]. Furthermore, research into the association between incarceration rates and reported drug use behavior suggests that correlations are weak at best [6, 7]. Still, sentencing laws continue to be used, and even broadened as means to deter illicit drug use.

In the early 1980s, cocaine grew in popularity in the US, with 1.6 million new users between 1982 and 1985, and a four-fold increase in cocaine-related emergency department visits between 1985 and 1988 [8, 9]. Public concern about cocaine and the emerging derivative crack (or base) cocaine (a less costly form of the drug that is smoked as opposed to inhaled) reached hysteria levels, with more than 1000 stories reported in various national newspapers and magazines in the months before the 1986 election [10]. Despite lack of scientific evidence, crack cocaine, in particular, was seen as a highly addictive drug that led to unpredictable, often violent behavior, including increased gang-related violence in urban areas [10–13]. In addition to media frenzy surrounding the wave of violence and addiction seen as stemming from the new form of cocaine, the widely-publicized death of Len Bias, a Boston Celtics draft pick who died following an overdose in his college dormitory solidified the public outcry for action from public officials [14–16]. This event, among others, ignited a political response to the growing epidemic which culminated in the enactment of the Anti-Drug Abuse Act (ADAA) of 1986, setting mandatory minimum sentences for illicit drug offenses.

The ADAA covered most illicit drugs and set differing minimum sentences based on type and quantity of these substances. The structure of the law was based in deterrence theory, which argues that human behavior is driven by cost-benefit ratios; therefore, increasing associated costs should deter a given behavior [17]. In the case of ADAA, this theory would suggest that drug use can be deterred to varying degrees based on the perceived harshness of criminal penalties [17].

In the 1980s, it was widely believed that cocaine base had more abuse potential and associated social harms relative to powder cocaine [18]. Therefore, the ADAA sentencing guidelines established harsher penalties for cocaine base than powder cocaine. Specifically, the quantity of substance that triggered the mandatory sentencing was a 100:1 ratio of powder cocaine to cocaine base, meaning that 500 g of powder cocaine would carry the equivalent criminal sentence to 5 g of cocaine base [18]. While this law was initially directed toward drug trafficking, it was amended in 1988 to include possession offenses [19].

In the two decades since enactment, evidence mounted that implementation of the ADAA led to significant racial disparities in incarceration [20]. This disparity appeared to be in part due to powder cocaine use being more prevalent among white males, while cocaine base use was more prevalent among black males. Black males were also more likely to be prosecuted to the fullest extent of the law than were white males [20, 21]. Although concern about the harms of cocaine base remained, efforts to reduce these racial disparities resulted in the Fair Sentencing Act of 2010 (FSA). This law decreased mandatory minimum sentences associated with cocaine base while maintaining existing sentences for powder cocaine. Specifically, the FSA modified the powder cocaine to cocaine base ratio from 100:1 to 18:1, so that 28 g of cocaine base held the same sentence as 500 g of powder cocaine [22].

While many studies have focused on the social consequences of mandatory minimum sentencing laws, surprisingly few have directly measured their impact on substance use in the general population [20, 21, 23–25]. In the little research that has been conducted, it was found that one of the unintended consequences of the ADAA was that the purity of cocaine increased by 57%, likely as a response to quantity restrictions [26], and thus it is possible that use of this drug also changed.

There are several historical examples of the impact of mandatory minimum sentences on drug use in the United States. In 1956, the Narcotic Control Act set such penalties for narcotics, including heroin; however, over the next decade, narcotic use accelerated and this legislation was considered a failure [27, 28]. As a result, these mandatory sentences were repealed in 1970 [27, 29]. However, some believed at the time that other types of drugs, especially cocaine, might have been more responsive to sentencing measures [28].

When enacted, mandatory minimum sentences were touted as a way to “curb drug use.” [30] The goal of this study is to assess the extent to which mandatory minimum sentences achieved the intended effect of deterring substance use behaviors by using the natural quasi-experiment of the introduction of mandatory minimum sentences for cocaine through the ADAA and their subsequent relaxation with the FSA. If mandatory minimum sentencing policies deterred cocaine use in the general population, then the ADAA should be associated with reduced use of both forms of the drug, with greater pressure applied to cocaine base. Second, if the sentence is the driving force behind cocaine base use, relaxing pressure on cocaine base use through the FSA should have resulted in increased use of cocaine base with no change in powder cocaine use. Finally, if any observed changes in drug use were indeed due to the ADAA and FSA, these laws should have had no influence on use of drugs not targeted by these policies (e.g., misuse of prescription medications like tranquilizers and sedatives). By including prescription drug misuse as a negative control, we can differentiate between changes in use of drugs explicitly included in the policy and changes in underlying trends independent of the sentencing guidelines, such as those that may be related to market factors or period effects (i.e., “Just Say No” campaign) [31].

## Methods

### Sample

Our sample is based on repeated cross-sectional data from the 1985, 1988, and 1990 National Survey on Drug Use and Health (NSDUH) for the ADAA cohort and the 2009, 2012, and 2013 surveys for the FSA cohort [32]. NSDUH, previously known as the National Household Survey on Drug Abuse, is a nationally-representative cross-sectional household survey sponsored by the Substance Abuse and Mental Health Services Administration (SAMHSA) [33]. Using a multi-stage area probability design, the survey gathers detailed information on tobacco, alcohol, and illicit drug use from households and individuals. The NSDUH pioneered several methodological innovations to enhance validity of the data, and reliability studies have shown consistent responses for lifetime and past year drug use [34]. In addition to NSDUH-specific validity reports, self-reported illicit drug use, in general, is considered a valid and reliable means of measurement [35, 36]. Response rates for these five years ranged from 77% in 1988 to 88% in 2009. The NSDUH is publicly available from SAMHSA and all participants provided informed consent.

We exclude children under the age of 15, since they received only a partial survey, as well as observations with missing information on cocaine base or powder cocaine use or a key covariate. We further exclude respondents who report having misused a prescription opioid (defined as having used an opiate for non-medical purposes, i.e. “to get high”) or used heroin in any form in the past year from both ADAA and FSA analyses in order to avoid potential confounding factors associated with the rising opioid epidemic. The final analytic sample size is 21,296 for the ADAA cohort and 130,574 for the FSA cohort reflecting the increase in the overall NSDUH sample between these time periods.

### Outcome measures

We define cocaine base as any freebase or crack cocaine use within the previous 12 months. The 1985 NSDUH survey was the first year that cocaine base was included in the questionnaire. In this survey, respondents were asked about any type of cocaine use in the past 12 months, and lifetime use of freebase cocaine. If the respondent reported having used any type of cocaine within the last year and freebase at any point, we categorized them as having used cocaine base within the last year. Although this may lead to overestimation of 1985 cocaine base use, this overestimation is likely minimal since this form of the drug did not emerge outside of select regions until roughly 1984 and is considered to have been widespread only beginning in 1985 [14, 37]. However, if there were an overestimation of cocaine base use in 1985, this would exaggerate our estimates in change of use of cocaine base following the ADAA, but should not affect the estimates for powder cocaine. For later surveys, respondents were directly asked about crack or freebase cocaine use within the last 12 months.

While crack and freebase cocaine are not identical forms of cocaine, both are considered cocaine base under the ADAA and FSA laws. For our ADAA models we use freebase use as our cocaine base indicator, since this terminology was most consistently understood during this period. While not all freebase users during this period reported crack use, all crack users reported freebase use. Over the FSA period, freebase use was no longer included in the survey since terminology had changed so that “crack” was the more recognizable term. Since definitions are consistent within time periods and, therefore, across grouped models, no biases should be introduced by the change in terminology.

Powder cocaine includes only those who have used powder cocaine within the past 12 months, but have not used cocaine base. If a person uses both forms of cocaine, they are categorized as cocaine base users; we conducted sensitivity analyses to assess the impact of this classification strategy (described in *Sensitivity Analyses* section).

Prescription drug misuse in the past 12 months is defined as use of prescription sedatives and/or tranquilizers (e.g., Quaalude, Xanax, or Valium) either without a prescription, in greater amounts than prescribed, more often than prescribed, or for any non-medical reason (i.e., to get high). Sedative and tranquilizer use was common in the 1960s and 1970s, and these medications were among the most commonly-prescribed psychoactive drugs in the US; both are associated with physiologic dependence and are considered potential drugs of abuse [38]. These drugs were selected because although the ADAA focused on cocaine base, it covered all commonly-used illicit drugs at the time, including marijuana, and no other campaigns contemporaneously targeted misuse of these substances. Narcotics or opiates are not included in our categorization of prescription medications of abuse due to their cyclic popularity and the potential confounding effects of the recent opioid epidemic. In fact, we exclude respondents who report past-year opioid use to ensure that confounders are not introduced. Opiates are further addressed through additional models described in the Sensitivity Analyses section below. The medications included in the prescription medication negative control were consistently assessed in all six survey years. By using prescription drugs as a negative control, we are able to isolate changes in cocaine use due to sentencing policies from temporal trends in drug use more generally.

### Study design

We model the ADAA and FSA separately, first to address differential implementation of mandatory minimum sentences by cocaine type in the ADAA, and then extend this analysis using the FSA to assess changes in drug use following relaxation of this sentence discrepancy. Three models per law are analyzed. The first model estimates the change in cocaine base use after law implementation. The second model estimates the change in powder cocaine use. If mandatory minimums drive the change in drug use, we would expect that use would decrease for both forms of cocaine, but that this decrease should be greater for cocaine base due to harsher penalties. Finally, to ensure that any change in drug use is in fact a result of sentencing guidelines, we fit a third model as a negative control: prescription drug misuse, indexed as misuse of tranquilizers and/or sedatives in the past year. Misuse of prescription medications was not targeted in either the ADAA or FSA laws, and thus should not have been affected by changes to sentencing guidelines for other drugs [36].

We use three years of survey data for both the ADAA and FSA models. As the ADAA was enacted in 1986, 1985 is considered the baseline year. Since drug use is defined as past year use, 1988 is used as the post-implementation year to allow for a full year of drug use after ADAA enactment. However, due to an amendment to the ADAA pertaining to possession sentencing in 1988, 1990 is also included to account for full implementation of the law (further described in the *Sensitivity Analyses* section). Similarly, the Fair Sentencing Act was enacted in August of 2010. Therefore, in order to obtain full-year drug use estimates after implementation, we use the 2012 NSDUH survey response. We further add 2013 responses to ensure robust estimation of post-FSA changes in drug use. As both the ADAA and the FSA were popular, bipartisan bills passed in election years, both laws were widely publicized as part of congressional campaigns and enacted immediately; therefore, two post-implementation years apiece should be sufficient to estimate changes in behavior related to sentencing reforms. We do conduct a sensitivity analysis around this time frame (as described in the *Sensitivity Analyses* section) and results are robust to additional years.

### Statistical analysis

We estimate the change in drug use using three identical multivariable logistic models per law, one for each drug. Drug use is defined as a binary indicator of having used within the past 12 months or not. All regressions are weighted to account for complex survey design in the standard errors. Using the principles described by Paternoster et al., a Z-test is used to compare coefficients across the models to determine if there is a differential impact of the law on cocaine base versus powder cocaine versus prescription drug misuse [39]. We use dummy variables for years to identify the likelihood of reporting drug use in the post-implementation period compared to the pre-implementation period. All models are adjusted for age (categorized as older or younger than 25 years to accommodate the oversampling of younger age groups), race (categorized as non-Hispanic white, non-Hispanic black, Hispanic, or other), gender, marital status (categorized as married, never married, and separated/widowed/divorced), education level (categorized as under 18, less than high school, high school graduate, some college, and college graduate), and income level (categorized by modified income quartiles). Models are also adjusted for past year use of licit (alcohol and tobacco) and other illicit (marijuana) drugs.

We use the Akaike information criterion for model selection and verified goodness of fit with a survey weight-adjusted Hosmer-Lemeshow test and Pregibon linktest. Since all three models had to be identical to allow for comparison, the three models had varying levels of fit. However, unadjusted simple logistic regressions of outcomes all indicated adequate specification. These estimates were nearly identical to adjusted regression estimates (available upon request).

Analyses were conducted with StataSE 14 statistical software using survey procedures to account for the study design. All reported *p*-values refer to two-tailed tests.

### Sensitivity analyses

Categorization of cocaine use: We re-categorized respondents who used both cocaine base and powder cocaine as powder cocaine users (*N* = 1489 in the ADAA cohort and *N* = 2847 in the FSA cohort), and find our results to be consistent regardless of categorization.

Modification of the ADAA: In 1988, the U.S. Congress modified the ADAA to include drug possession in sentencing guidelines in addition to drug trafficking. In our main analysis, we use 1990 as the post-implementation year to ensure we are capturing full implementation. However, we estimate the change in drug use in 1988 as well to monitor any variation in use that might be present as the law is expanded and revised. We also conduct an analysis including 1991 in the post-implementation period, which again provides similar estimates to the main analysis. 

Opiate users: To account for possible substitution effects of users switching from one drug, whether cocaine or other prescription medications, to opioids, we exclude respondents with non-medical use of prescription opioids or who used heroin in any form in the past year from both ADAA and FSA analyses. However, to ensure that these respondents are not fundamentally different, due to potential differences in level of addiction or dependence or risk seeking behavior, we perform a sensitivity analysis including these respondents. While individual model estimates varied somewhat from primary model estimates, overall conclusions remained consistent.

## Results

**Fig. 1:**
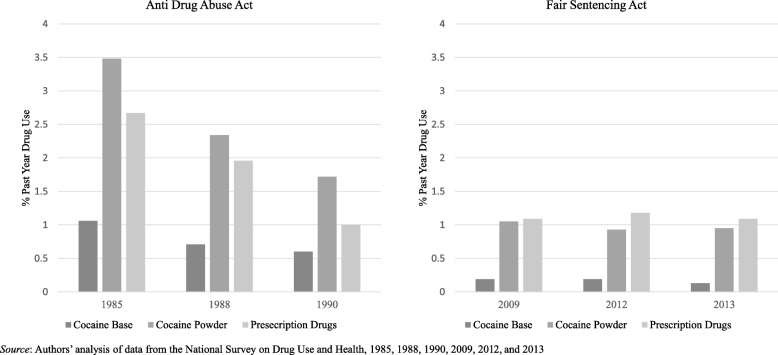
Drug use trends during the ADAA and FSA periods

Table 1 shows the distribution of sample characteristics by survey year. Educational attainment is higher in the more recent cohort, with nearly a quarter of respondents reporting less than a high school education during the ADAA cohort as compared to approximately 14% of the FSA cohort. In general, past-year use of cocaine base was rare in the study sample, with just 1% reporting use in the ADAA cohort and even fewer, less than 0.05%, in the FSA cohort. Powder cocaine and prescription drug misuse were somewhat more common with 2 to 4% of sample respondents reporting use among the ADAA cohort and about 1% in the FSA cohort. Use of all three drugs of interest declined over the ADAA period, but reported use was higher in the ADAA period than in the FSA period. Overall drug use trends over the ADAA and FSA periods are shown in Fig. 1.

**Table 1:** Sociodemographic characteristics of survey participants during ADAA and FSA periods

	ADAA						FSA					
	1985		1988		1990		2009		2012		2013	
	*n* = 6456 [*N* = 171,780,375]		*n* = 6948 [*N* = 181,588,628]		*n* = 7892 [*N* = 186,296,389]		*n* = 43,227 [*N* = 228,441,140]		n = 43,684 [*N* = 236,094,646]		n = 43,663 [*N* = 239,616,555]	
	%	(SE)	%	(SE)	%	(SE)	%	(SE)	%	(SE)	%	(SE)
Younger than 25 years old	22.47%	(0.007)	20.72%	(0.007)	20.02%	(0.006)	18.19%	(0.002)	18.22%	(0.002)	18.20%	(0.002)
Female	53.53%	(0.010)	52.32%	(0.008)	52.49%	(0.009)	51.94%	(0.004)	52.08%	(0.004)	51.96%	(0.005)
Race												
Non-Hispanic White	80.47%	(0.011)	79.53%	(0.014)	78.84%	(0.016)	67.46%	(0.004)	65.64%	(0.004)	65.22%	(0.004)
Non-Hispanic Black	10.85%	(0.007)	10.99%	(0.011)	10.98%	(0.013)	11.99%	(0.003)	11.70%	(0.003)	11.92%	(0.003)
Hispanic	6.79%	(0.007)	7.27%	(0.008)	7.54%	(0.009)	14.15%	(0.003)	15.12%	(0.003)	15.31%	(0.003)
Other	1.90%	(0.003)	2.20%	(0.003)	2.65%	(0.004)	6.40%	(0.002)	7.54%	(0.003)	7.55%	(0.003)
Marital status												
Married	60.38%	(0.009)	60.37%	(0.010)	61.15%	(0.011)	52.83%	(0.004)	50.89%	(0.004)	50.36%	(0.004)
Separated/divorced/widowed	16.19%	(0.007)	16.83%	(0.006)	16.74%	(0.008)	18.06%	(0.004)	19.55%	(0.004)	19.47%	(0.004)
Never married	23.44%	(0.007)	22.81%	(0.008)	22.11%	(0.008)	29.10%	(0.003)	29.57%	(0.003)	30.16%	(0.003)
Education												
15–18 year olds	5.76%	(0.003)	5.53%	(0.002)	5.31%	(0.002)	5.19%	(0.001)	5.01%	(0.001)	4.93%	(0.001)
Less than high school	23.61%	(0.010)	24.30%	(0.011)	22.74%	(0.010)	14.29%	(0.003)	13.68%	(0.003)	12.64%	(0.003)
High school graduate	35.18%	(0.010)	34.34%	(0.010)	34.22%	(0.010)	29.10%	(0.004)	28.11%	(0.004)	28.09%	(0.004)
Some college	18.29%	(0.008)	18.87%	(0.009)	18.67%	(0.008)	23.68%	(0.004)	24.90%	(0.004)	25.59%	(0.004)
College graduate	17.15%	(0.011)	16.96%	(0.009)	19.06%	(0.011)	27.74%	(0.004)	28.30%	(0.004)	28.75%	(0.004)
Annual Income (quartiles)												
0–25%	17.17%	(0.009)	17.73%	(0.009)	22.04%	(0.010)	17.15%	(0.003)	18.62%	(0.003)	18.00%	(0.003)
25–50%	21.58%	(0.007)	17.88%	(0.008)	16.64%	(0.006)	21.64%	(0.004)	22.10%	(0.004)	20.49%	(0.003)
50–75%	29.72%	(0.009)	28.76%	(0.010)	23.60%	(0.008)	28.37%	(0.004)	27.24%	(0.004)	28.04%	(0.004)
75–100%	26.69%	(0.011)	30.61%	(0.012)	31.04%	(0.011)	32.83%	(0.004)	32.04%	(0.004)	33.47%	(0.004)
Unknown	4.84%	(0.004)	5.01%	(0.006)	6.68%	(0.007)						
Alcohol Use in Past Year	74.29%	(0.013)	69.72%	(0.012)	67.49%	(0.015)	68.69%	(0.004)	68.36%	(0.004)	68.26%	(0.004)
Cigarette Use in Past Year	35.88%	(0.009)	34.38%	(0.009)	32.32%	(0.009)	27.04%	(0.004)	25.61%	(0.004)	24.84%	(0.004)
Marijuana Use in Past Year	12.94%	(0.005)	9.50%	(0.005)	9.34%	(0.005)	9.64%	(0.002)	10.69%	(0.002)	11.23%	(0.002)

*Notes: SE* standard error

As expected, alcohol, cigarette and marijuana use were highly predictive of both powder and base cocaine use as well as prescription drug abuse. Race was also highly predictive of the type of drug used, as noted in prior literature on racial disparities in drug-offense related incarceration [23]. In both the ADAA and FSA models, non-Hispanic blacks have a lower probability of reporting powder cocaine use or prescription drug misuse than non-Hispanic whites, but are significantly more likely to report cocaine base use (odds ratio (OR_ADAA_): 2.59, standard error (SE): 0.54; OR_FSA_: 2.58, SE: 68).

As shown in Table 2, use of powder cocaine decreased after implementation of the ADAA while cocaine base use remained largely unchanged (OR_powder_: 0.59, SE: 0.08). While this may appear to suggest that the ADAA was effective in deterring some form of cocaine use, when we look at the change in prescription drug misuse, we again find a significant decline (OR_rx_: 0.42, SE: 0.08). When we compare the magnitude of decline in use across all three drugs using Z-tests, we find that all drugs declined to similar degrees, showing no differential decline by drug or form of cocaine (Z-score_base vs powder_: 0.80, *P* = 0.42; Z-score_base vs rx_: 1.94, *P* = 0.05; Z-score_powder vs rx_: 1.50, *P* = 0.13). If any indication of differential change in use is suggested in these models, it is that prescription drug use declined to a greater extent than cocaine base, directly contradicting the hypothesis of deterrence.

**Table 2:** Change in drug use post-ADAA and post-FSA by form and type of drug

	Cocaine base		Powder cocaine		Prescription drugs	
	Odds ratio	(SE)	Odds ratio	(SE)	Odds ratio	(SE)
ADAA (N = 21,296)						
Expansion of ADAA (1988)	0.85	(0.174)	0.83	(0.112)	0.84	(0.132)
Fill ADAA Implementation (1990)	0.72	(0.153)	0.59	(0.080)***	0.42	(0.078)***
FSA (N = 130,574)						
FSA Implementation (2012, 2013)	0.75	(0.167)	0.81	(0.074)**	1.00	(0.091)

*Notes: SE* standard error, *** *P* < 0.01, ** *P* < 0.05, * *P* < 0.1; all analyses excludes person who used opiates in the past year, adjusted for age, sex, race/ethnicity, marital status, education, income, alcohol use, tobacco use, and marijuana use; all models are weighted to account for complex survey design

**Fig. 2:**
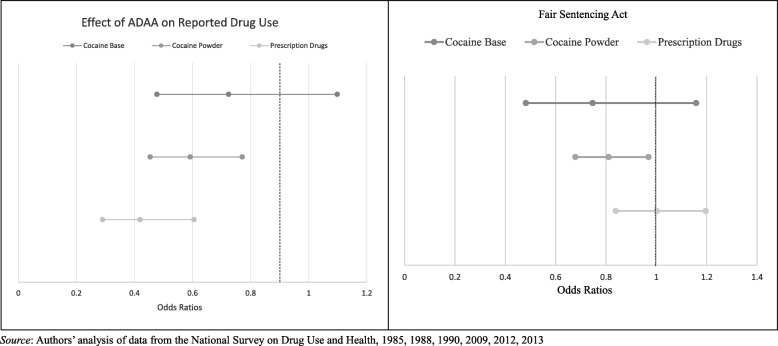
Change in reported drug use after implementation of and relaxation of mandatory minimum sentencing policies

In the FSA models, we expected to see no change in use of powder cocaine and misuse of prescription drugs since neither were affected by the law. However, while we found no change in prescription drug misuse, we again found a decrease in powder cocaine use after FSA implementation (OR_powder_: 0.81, SE: 0.07. While we expected to find an increase in cocaine base use due to relaxation of sentencing, instead we found no change in cocaine base use (OR_base_: 0.75, SE: 0.17). In comparing the magnitude of the change across the three types of drugs, we again find no statistically different changes between drug forms. This is contrary to expected findings if sentencing guidelines are driving change in substance use. Differential changes in drug use for the ADAA and FSA are represented in Fig. 2.

## Discussion

The ADAA set different mandatory minimum sentencing guidelines to deter the use of cocaine base more than powder cocaine. Based on our results, it can be concluded that these differential sentences are not associated with differing drug use behavior. Our study does not propose to identify other explanations for changes in drug use patterns, such as substitution, availability, purity or cost, and therefore, we cannot rule out that these occurred. Instead, this study narrowly focuses on mandatory sentencing laws to determine whether or not drug use patterns are consistent with those expected to occur if harsher sentences deterred use. Here, we find no evidence that sentencing policies deterred cocaine base use. These findings are consistent with prior research that has found that incarceration rates are unrelated to drug use behavior and that effectiveness of a deterrence tends to hinge on certainty and immediacy of punishment rather than severity [6, 40].

Mandatory minimum sentencing laws for trafficking and possession were not associated with differential changes in drug use for crack as compared to powder cocaine use, as would have been expected if sentencing guidelines drove change in drug use. While there was a statistically significant decline in powder cocaine after the ADAA was fully enforced, this decline did not differ from the decline in prescription drug misuse which was unrelated to mandatory sentencing implementation, and no decline was seen in cocaine base, the primary drug of focus. Our findings suggest that drug use trends shifted alongside, rather than in direct response to, implementation of the ADAA and FSA. In short, the decline in cocaine base and powder cocaine use that occurred in the US during the 1980s cannot be attributed to the ADAA. Similarly, the FSA is not associated with changes in cocaine base use despite relaxing sentencing requirements for cocaine base. These findings do not support the hypothesis that the ADAA had a substantive influence on use of cocaine base and powder cocaine in the community-dwelling population. In total, we find no evidence to support mandatory minimum sentences as a causal explanation for changes in drug use behavior, and in fact, data is inconsistent with this hypothesis.

It may be argued that no differential effect in drug use is seen because while cocaine base users experienced harsher penalties, other drug users may have been more readily able to decrease use because they were less addicted. Despite harsh penalties, cocaine base users simply had a more inelastic demand for their drug of choice compared to other drug users, so even less severe consequences resulted in larger changes in behavior among powder cocaine and those who misuse prescription drug. While this scenario could result in all three categories of drug users decreasing use at similar rates, this explanation has no physiological basis. Although some reports in the 1980s suggested that cocaine base is more addictive than other forms of cocaine, these claims have largely been shown to be false [41, 42]. In fact, there is little scientific evidence suggesting that cocaine base is substantially chemically different from other forms of cocaine [10, 42, 43]. Physiologically, deterrence mechanisms, such as sentencing policies, should have similar effects on powder cocaine and cocaine base users. Furthermore, more recent research suggests that 85% of cocaine users have not developed dependence after 10 years of use, indicating that dependence is likely not a major factor in trends in use [41]. To the extent that other behavioral characteristics result in more inelastic demand among cocaine base users compared to other drug users, of the establishment of mandatory minimum sentences was insufficient to change use at the population level – ultimately resulting in the ineffectiveness of these policies.

Measurement error, whether due to social desirability (resulting in underestimation of illicit activities), high rates of incarceration of illicit drug users (who were thus ineligible for the NSDUH), or our baseline definition of cocaine base use, should have led to an exaggeration of the decline in cocaine base use compared to our negative control of prescription drug misuse. As a result, our null findings for the ADAA are undoubtedly conservative as this measurement error should have exaggerated any effects of the law if they had been present. Findings from the FSA cohort further suggest that mandatory minimums did not impact cocaine use. If this law had directly influenced cocaine use, the expected result would have been an increase use of in cocaine base with the relaxation of sentencing penalties. In contrast, there was again no change in use, with powder cocaine, a form unaffected by the FSA declining in use.

There are several potential explanations for these findings. Increasing awareness of the harms associated with drug use – whether through formal awareness campaigns, news reporting or local community efforts – occurred alongside these criminal law changes. [44]. Relative cost or availability of other substances may have led to substitution (i.e., use of marijuana or heroin instead of cocaine) [45]. Availability and cost of cocaine itself may also be a contributing factor. In fact, other studies exploring causes of the decreased cocaine consumption in the 2000s have found that supply-side factors may have contributed to changing cocaine use behavior in the United States [6, 45]. A variety of global economic issues, increased seizures and reduced production prevented easy importation while purity-adjusted price increased by more than 40% [45–47]. Other drug-specific trends may also contribute to our findings. The popularity of different substances varies over time. For instance, the rising prescription opioid epidemic that began in the 2000s may have been a contributing factor to lower cocaine use in the post-FSA period [48]. Future research should assess opioid use (both prescription and heroin) as a way of managing highs when other drugs are not available or are less popular. This study should be seen as aiming to rule out mandatory minimum sentencing policies as the explanation for the decline in cocaine base use, but not as providing an alternative explanation. These findings need to be replicated in other US data and in other countries that have reformed sentencing guidelines for drug offenses.

### Limitations

There are several limitations of the NSDUH survey data in estimating changes in drug use behavior. First, while drug use is self-reported, the reliability and validity of the NSDUH measures have been demonstrated repeatedly [34–36, 49]. However, to the extent that social desirability is present, we would expect that after implementation of more severe criminal penalties, cocaine use would be differentially under-reported, contrary to these findings. Second, only non-institutionalized civilians (e.g. non-incarcerated, or not in a nursing facility or other institutionalized setting) are eligible for the NSDUH. Therefore, if cocaine users were incarcerated at higher rates after implementation of the law, we would again expect to see larger decreases in cocaine users. As we did not observe a significant decrease in cocaine use post-ADAA, this suggests that these biases did not substantially influence our results. It is possible that the wide reach of the ADAA resulted in a spillover effect into other substances, such as prescription drugs, that were not addressed by the law. However, while a spillover effect may contribute to the decline in prescription drug misuse following the ADAA, it does not explain the continued decrease in prescription drug use following the FSA. Finally, we were unable to account for state-level policies if enacted during that time that may have modified the effect of federal policies in particular areas. However, a literature review suggests that the ADAA held popular bipartisan appeal nationwide at the time of enactment, as did the FSA, so that states were not inclined to pass legislation that may counteract the potency of either sentencing reform in the years immediately before or after the federal reforms [16]. Additionally, even if states chose to enact reforms, they would have been required to be at least as restrictive or generally similar to federal law [50]. Furthermore, recent evidence has found that while states incarcerate for drug offenses at varying rates, incarceration rates are not associated with reduced drug use [7].

## Conclusions

NSDUH is the largest and most valid source of data on drug use in the US population and uses standardized methods for ensuring accuracy of assessment of illicit activities. Our quasi-experimental study design of examining the imposition, then subsequent relaxation, of criminal sentencing penalties allowed for a rigorous assessment of the impact of mandatory minimum sentencing laws on cocaine use in the population. By including prescription tranquilizer/sedative misuse as a negative control, we added an additional layer of control to more precisely identify changes in drug use related to sentencing policies.

In sum, we found no direct correlation between mandatory minimum sentencing laws on cocaine use. These findings are consistent with the small body of extant literature on mandatory minimum sentencing policies on narcotic use in the mid-twentieth century. We do not, however, assume to rule out other causes of the decline in cocaine use, but merely state that findings do not support the claim that mandatory minimum sentencing laws deter drug use. The current opioid epidemic has somewhat different challenges than the cocaine epidemic examined in this study (i.e., opioid misuse and dependence often occurs subsequent to a legal prescription to manage pain); therefore, we cannot directly comment on the implications of our findings on opioid misuse. However, taken together with prior research, there is little evidence that mandatory minimum sentencing laws deter drug use behavior of cocaine or other illicit substances. Policymakers aiming to reduce substance misuse should instead consider evidence-based program options for prevention or treatment [51, 52].

## Abbreviations

ADAA: Anti-Drug Abuse Act
FSA: Fair Sentencing Act
NSDUH: National Survey on Drug Use and Health
OR: Odds ratio
SAMHSA: Substance Abuse and Mental Health Services Administration
SE: Standard error
US: United States
